# lncRNA ADAMTS9-AS1/circFN1 Competitively Binds to miR-206 to Elevate the Expression of ACTB, Thus Inducing Hypertrophic Cardiomyopathy

**DOI:** 10.1155/2022/1450610

**Published:** 2022-03-31

**Authors:** Wei Feng, Shuo Han

**Affiliations:** ^1^Department of Ultrasound, The Fourth Affiliated Hospital of China Medical University, Shenyang 110032, China; ^2^Department of Cardiology, The Fourth Affiliated Hospital of China Medical University, Shenyang 110032, China

## Abstract

Hypertrophic cardiomyopathy (HCM) is a genetic cardiac disease and can result in substantial disability. The current study explored the potentials of long noncoding RNA- (lncRNA-) circular RNA- (circRNA-) microRNA- (miRNA-) messenger RNA (mRNA) networks in HCM. Firstly, HCM-related microarray data were procured from the GEO database, with differentially expressed genes (DEGs) obtained. HCM-related target genes were retrieved in combination with GeneCards and CTD databases, and candidate target genes were subsequently obtained by intersection screening. Further, an interaction network diagram of candidate target genes was constructed using the STRING database, and the hub genes in the network were determined according to the core degree. The “ClusterProfiler” package of the R software was adopted for GO and KEGG analyses of candidate target genes, to analyze the potential molecular pathways in HCM. Next, upstream miRNA, lncRNA, and circRNA of ACTB were predicted with RNAInter, mirDIP, TargetScan, DIANA-LncBase, and StarBase databases, followed by construction of lncRNA/circRNA-miRNA-mRNA coexpression networks. ACTB, miR-206, circFN1, and ADAMTS9-AS1 expression in peripheral blood samples from HCM patients and normal healthy controls were detected using RT-qPCR. Moreover, rat cardiomyocyte cell lines H9c2 and HEK293 cells were selected for *in vitro* verification of competitive endogenous RNA (ceRNA) regulation mechanism. A total of 15 candidate target genes related to HCM were screened using the online databases. Further protein-protein interaction analysis identified ACTB as the hub gene for HCM. The targeted binding relationship between miR-206, miR-145-5p, miR-1-3p, and ACTB was found. Furthermore, ADAMTS9-AS1 and circFN1 were discovered as the upstream genes of miR-206. Moreover, ADAMTS9-AS1, circFN1, and ACTB were found to be poorly expressed, and miR-206 was highly expressed in HCM. *In vitro* experimentation further confirmed that ADAMTS9-AS1 and circFN1 could competitively bind to miR-206, thereby augmenting ACTB expression. Taken all, ADAMTS9-AS1/circFN1-miR-206-ACTB regulatory network may involve in HCM occurrence, providing a novel theoretical basis for in-depth understanding of mechanism of HCM.

## 1. Introduction

Hypertrophic cardiomyopathy (HCM), a heterogeneous disorder of genetic disposition, is usually induced by sarcomere mutations, which can lead a number of adverse events, including fibrosis, hypercontraction, left ventricular hypertrophy, and reduced compliance [1]. An increase in myofilament contractility is regarded as one of the key factors implicated in the pathogenesis of hypertrophic cardiomyopathy [2]. Fortunately, the advent of implantable cardioverter-defibrillators combined with mature risk stratification algorithms has transformed the management strategies for HCM, making the possibility of sudden death prevention a reality [3]. Nevertheless, in spite of the enhanced understanding of causal mutations, the underlying pathway that leads to cardiomyopathies from genetic defects remains complex and involves other diseases and thus requires further exploration [4].

The beta-actin gene (ACTB) is implicated in the process of vascular remodeling and further leads to cardiovascular diseases [5]. It is also noteworthy that ACTB is differentially expressed in a plethora of cardiovascular diseases, including HCM [6]. Moreover, a prior study documented that ACTB was underexpressed in homocysteine-treated human umbilical vein endothelial cells [7]. In addition, a number of researches have explored the roles of microRNA (miRNA), long noncoding RNA (lncRNA), circular RNA (circRNA) regulation in cardiovascular-related diseases, and abnormal expressions of the aforementioned have been detected in myocardial tissues and peripheral blood samples of patients with varying heart diseases [8, 9]. Interestingly, increased expressions of one such miRNA, namely, miR-206, were previously shown to trigger cardiac hypertrophy and inhibit death of cardiomyocytes [10], while studies have also come across increased plasma levels of miR-206 in patients with inflammatory cardiomyopathy [11]. Initial prediction results from the StarBase database further predicted that lncRNA ADAMTS9-AS1 (ADAMTS9-AS1) and circular RNA FN1 (circFN1) served as the upstream genes of miR-206. Furthermore, lncRNAs are further known to play an important role in the regulation of gene expression by functioning as ceRNAs [12]. Moreover, a prior study indicated the important roles of ADAMTS9-AS1 in different cancer types, including prostate cancer, breast cancer, and hepatocellular carcinoma [13–15]. However, its role in cardiovascular diseases remains unclear. Meanwhile, the FN1 pathway is well-established to exert significant effects on myocardial fibrosis, aiding the determination of dilated cardiomyopathy etiology and providing a potential therapeutic target [16]. Besides, the level of circFN1 was previously illustrated to be diminished in the context of mouse diabetic cardiomyopathy [17]. Therefore, the current study is aimed to explore the possible molecular mechanism of the lncRNA/circRNA-miRNA-mRNA coexpression regulatory network involved in HCM.

## 2. Materials and Methods

### 2.1. Ethics Statement

The current study was approved by the Ethics Committee of The Fourth Affiliated Hospital of China Medical University and conducted strictly in accordance with the *Declaration of Helsinki.* Signed informed consents were obtained from all participants prior to specimen collection.

### 2.2. Acquisition and Analysis of Microarray Data at Gene Expression Omnibus (GEO)

Firstly, whole transcriptome microarray datasets GSE36961, GSE148602, and GSE68316 were retrieved from the GEO database (https://www.ncbi.nlm.nih.gov/gds) by searching “hypertrophic cardiomyopathy” with study type as “Expression profiling by array” and Organisms as “Homo sapiens.” The GSE36961 dataset comprised of 106 tissue samples from HCM patients obtained by myomectomy and 39 heart tissue samples obtained from control donors. Meanwhile, the GSE148602 dataset included 15 peripheral blood samples from HCM patients and 7 normal control samples, whereas the GSE68316 dataset included myocardial tissue samples from 7 HCM patients and 5 healthy individuals.

R language “limma” package (http://www.bioconductor.org/packages/release/bioc/html/limma.html) was adopted to identify the differentially expressed genes (DEGs) in the two datasets, with a *P* value < 0.05 as the threshold. The R language “ggplot2” package was utilized to draw volcano plots. Simultaneously, the R language “corrplot” package was used to perform correlation analysis on the mRNA expression of candidate genes.

### 2.3. Disease-Related Database Search

HCM-related target genes were retrieved through the GeneCards databases (https://www.genecards.org/) and Comparative Toxicogenomics Database (CTD) (http://ctdbase.org/). The retrieval time was March 10, 2021 (due to database update, the retrieval results could be varying at different times). The screening condition of GeneCards database was a relevance score ≥ 5, and the screening condition of CTD database was set as the inference score ≥ 20.

### 2.4. Functional Enrichment Analysis of Candidate Genes

The Venn Diagram tool (http://bioinformatics.psb.ugent.be/webtools/Venn/) was adopted to identify the intersection of the results from microarray data analysis and from GeneCards and CTD database retrieval in order to obtain a set of candidate genes. The obtained candidate genes were subjected to Gene ontology (GO) and Kyoto Encyclopedia of Genes and Genomes (KEGG) enrichment analysis using the R language “ClusterProfiler” package (http://www.bioconductor.org/packages/release/bioc/html/clusterProfiler.html) to analyze the cell functions and signal pathways primarily affected by potential targets and key targets. The criterion for statistical significance was set at *P* < 0.05.

### 2.5. Construction of Protein Interaction Network of Candidate Gene

The interaction network of target genes was obtained through the STRING database (https://string-db.org), and the species condition was limited to “Homo sapiens.” Subsequently, a regulatory relationship network was constructed, and the results of the network diagram were analyzed and sorted using the Cytoscape (v3.6.0) software. The degree value and combine score value was represented by color. Lastly, the candidate genes were ranked according to the degree value (core level).

### 2.6. Prediction of Upstream miRNA/lncRNA/circRNA of Candidate Genes

The RNAInter (http://mirwalk.umm.uni-heidelberg.de/), mirDIP (http://diana.imis.athena-innovation.gr/DianaTools/index.php?r=microtv4/index), and TargetScan (http://www.TargetScan.org/mamm_31/) databases were adopted to predict the upstream miRNA of candidate target genes (mRNA). Subsequently, the Draw Venn Diagram tool was employed to intersect the obtained results. DIANA-LncBase (http://carolina.imis.athena-innovation.gr/diana_tools/web/index.php?r=lncbasev2/index-predicted) and StarBase databases (http://starbase.sysu.edu.cn/starbase2/) were then used to predict the upstream lncRNA and circRNA of miRNA, respectively. Afterwards, the lncRNA/circRNA-miRNA-mRNA coexpression regulation network was constructed, and the Cytoscape software (v3.6.0) was employed to visualize the picture.

### 2.7. Clinical Sample Collection

A total of 24 patients with HCM admitted at The Fourth Affiliated Hospital of China Medical University were included in the current study, and 24 normal healthy participants who were at The Fourth Affiliated Hospital of China Medical University for physical examination during the same period were selected as the control group. Initially, 5 mL of peripheral blood samples was collected from the subjects and immediately stored in a refrigerator at -80°C for subsequent experimentation.

### 2.8. Cell Culture

Rat cardiomyocyte cell lines H9c2 and HEK293 were both purchased from American Type Culture Collection (ATCC) and cultured in Dulbecco's modified Eagle's medium (Beijing Solarbio Science & Technology Co., Ltd. Beijing, China) containing 10% fetal bovine serum in a humidified incubator at 37°C with 5% CO_2_ in air.

### 2.9. Reverse Transcription Quantitative Polymerase Chain Reaction (RT-qPCR)

Total RNA content was extracted from the peripheral blood of healthy participants and patients with the help of TRIzol kits (Themo Fisher Scientific, Carlsbad, California, USA). According to the instructions of Taqman MicroRNA Assays Reverse Transcription Primer (4427975, Applied Biosystems, Foster City, California, USA), the obtained RNA was reverse-transcribed into cDNA. Reverse transcription reaction conditions were 37°C for 30 min or 85°C for 5 s. Subsequently, 5 *μ*L each of the above cDNA product was taken as a template for PCR amplification. The PCR reaction system was as follows:5 *μ*L reverse transcription product, 13 *μ*L 2× QuantiTect SYBR Green RT-PCR Master Mix, 10 *μ*mol/*μ*L upstream primer and downstream primer (each 0.5 *μ*L), and 6 *μ*L DNAase-free water, for a total of 25 *μ*L. The mRNA/lncRNA/circRNA reaction conditions were as follows: at 95°C for 5 min, at 95°C for 20 s, at 60°C for 1 min, and at 72°C for 30s, with a total of 45 cycles. The miRNA reaction conditions were as follows: at 95°C for 10 min, followed by a 2-step PCR program of 95°C for 15 s, and 60°C for 1 min for 40 cycles. U6 was employed as the internal reference for miRNAs, while others used glyceraldehyde-3-phosphate dehydrogenase (GAPDH) as the internal reference. The 2^-*ΔΔ*CT^ method was adopted to measure target gene expression. The primers used are displayed in Table S1.

### 2.10. Dual-Luciferase Reporter Gene Assay

A dual-luciferase reporter gene assay was carried out to verify whether ACTB served as a direct target gene of miR-206. The 3′untranslated region (UTR) gene fragment of ACTB gene was cloned and amplified, and then, the PCR product was cloned into a polyclonal site downstream of Luciferase gene in pmirGLO (ArtNo. E1330, Promega Corporation, Madison, WI, USA) and regarded as pACTB-WT. Subsequently, the binding sites of miR-206 predicted by bioinformatics were subjected to site-specific mutagenesis to construct a pACTB-MUT vector. The pRL-TK vector expressing luciferase (ArtNo. E2241, Promega Corporation) was employed as the internal reference. miR-206-mimic and negative control (NC) were cotransfected with luciferase reporter vectors into HEK293 cells, and dual-luciferase activity was then detected according to the method provided by Promega Corporation. The same method was adopted to verify the targeted relationship between ADAMTS9-AS1 and miR-206 as well as between circFN1 and miR-206.

### 2.11. RNA Immunoprecipitation (RIP) Assay

Magna RIP TM RNA binding protein immunoprecipitation kits (Millipore Corp., Billerica, MA, USA) were employed for RIP assay. Briefly, H9c2 cells were lysed in complete RNA lysis buffer, and then, RIP buffer containing immunoglobulin G (IgG) or Argonaute 2 (AGO2) antibody (mouse, Millipore Corp.) coupled magnetic beads was added to the cell lysis buffer. Subsequently, the lysate was incubated overnight. On the following day, the lysate was incubated with protease K for 30 min, followed by extraction of the immunoprecipitation RNA. Finally, the expression patterns of ADAMTS9-AS1, circFN1, and miR-206 were detected by RT-qPCR and agarose gel electrophoresis.

### 2.12. RNA FISH

Cultured H9c2 cells were seeded in a 12-well plate, and the plate was taken out for treatment when H9c2 cells reached around 80% confluence. The culture medium of each well was absorbed, and the cells were fixed with 4% paraformaldehyde at room temperature for 20 min, cleared with xylene for 3 times, 5 min each, and then hydrated twice with ethanol (100%), 2 min each. Subsequently, the cells were treated with protease K detachment solution, rehydrated in 2× SSC, hybridized with 10 *μ*L of probe, and incubated at 37°C for 12 h to 16 h. The cells were washed after hybridization, washed twice with 2× SSC (37°C) for 10 min each time, and then incubated with 30 *μ*L-60 *μ*L rhodamine anti-digoxin antibody or fluorescein Isothiocyanate (FITC) at room temperature for 20 min. Following another wash, the cells were incubated with 30 *μ*L-60 *μ*L anti-ovalbotin antibody at room temperature for 20 min, stained with 10 *μ*L-20 *μ*L 4′,6-diamidino-2-phenylindole, and then observed under a fluorescence microscope as soon as possible or stored in a refrigerator at -20°C in an enclosed box.

### 2.13. Statistical Analysis

Statistical analyses were performed using the SPSS 21.0 version (IBM, Armonk, New York, USA). Measurement data were expressed by mean ± standard deviation. Independent sample *t* test was conducted for comparisons between two groups. One-way analysis of variance (ANOVA) was adopted for multiple group comparisons, followed by Tukey's post hoc test. Pearson correlation was employed to analyze the correlation between circFN1 and ACTB, ADAMTS9-AS1 and ACTB, and miR-206 and ACTB. A value of *P* < 0.05 was regarded statistically significant.

## 3. Results

### 3.1. Identification of 15 Candidate Genes Related to the Occurrence of HCM

**Figure 1:**
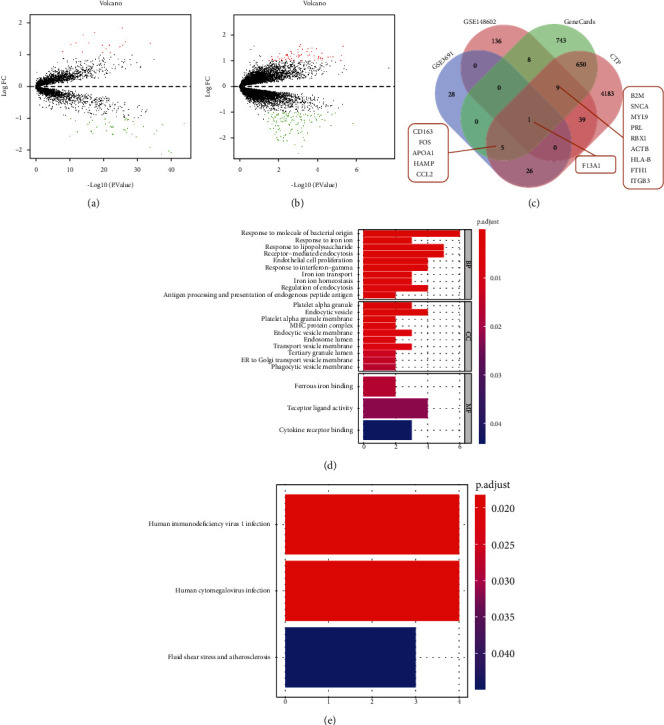
The screening and functional enrichment of candidate genes related to the occurrence of HCM. (a, b) Differentially expressed mRNA in HCM samples and normal samples in GSE36961 and GSE148602 datasets. Green dots indicate downregulation, red dots indicate upregulation, gray dots indicate no significant difference, and the horizontal axis represents the logarithmic value of the multiple (FC) of the difference between different groups at the base of 2, log_2_ (FC), and the vertical axis represents the negative logarithmic value of the *P* value for the significance test of the difference, which was -log10 (*P* value). (c) The Venn diagrams of the DEGs from the GSE36961 and GSE148602 datasets and the search results from GeneCards and CTD databases. (d) GO function analysis of 15 candidate DEGs at the levels of biological process (BP), cell components (CC), and molecular function (MF). (e) KEGG pathway analysis of the 15 candidate DEGs. The size of the dot indicates the number of selected genes, and the color indicates the *P* value of the enrichment analysis.

Firstly, we set out to screen the key factors related to the pathogenesis of HCM and combine the results with online databases to construct a lncRNA/circRNA-miRNA-mRNA coexpression network (ceRNA network) to further explore the biological functions of the ceRNA network in the occurrence of HCM, aiming to provide a theoretical basis for finding HCM blood circulation markers. Figure S1 shows the bioinformatics screening of key genes for HCM. Initially, we obtained 60 DEGs from the GSE36961 dataset, among which 17 were upregulated and 43 were downregulated (Figure 1(a)). Meanwhile, analyses of the GSE148602 dataset reared 193 DEGs, wherein 67 were upregulated and 126 were downregulated (Figure 1(b)). Additionally, HCM-related genes were retrieved from the GeneCards database, and 1416 genes were obtained using relevance score ≥ 5 as the screening threshold. Besides, after combination of CTD database, 4913 genes were obtained using inference score ≥ 20 as the screening threshold. DEGs from the GSE36961 and GSE148602 datasets were overlapped and intersected with the retrieval results of GeneCards and CTD databases to screen 15 candidate DEGs (Figure 1(c)). It should be noted that the screening of candidate DEGs was validated to meet the overlapping times of three or more times in GSE36961, GSE148602, GeneCards, and CTD, which also ensured the screening conditions of key genes: differential expression in clinical myocardial tissue or blood samples and literature evidence.

Afterwards, GO and KEGG analyses were performed on the 15 candidate DEGs. GO functional analysis illustrated that the 15 candidate DEGs were primarily enriched in response to molecule of bacterial origin (GO:0002237), in response to iron ion (GO:0010039), in response to lipopolysaccharide (GO:0032496), and receptor-mediated endocytosis (GO:0006898) in the biological process (BP). Meanwhile, in the cell components (CC), DEGs were mainly enriched in platelet alpha granule (GO:0031091), endocytic vesicle (GO:0030139), and platelet alpha granule membrane (GO:0031092). With regard to molecular function (MF), DEGs were primarily enriched in ferrous iron binding (GO:0008198), receptor ligand activity (GO:0048018), and cytokine receptor binding (GO:0005126) (Figure 1(d)). Moreover, KEGG pathway analysis revealed that the 15 candidate DEGs were chiefly enriched in human immunodeficiency virus 1 infection (hsa05170), human cytomegalovirus infection (hsa05163), and fluid shear stress and atherosclerosis (hsa05418) entries (Figure 1(e)).

### 3.2. ACTB May Be the Key Gene Involved in the Occurrence of HCM

**Figure 2:**
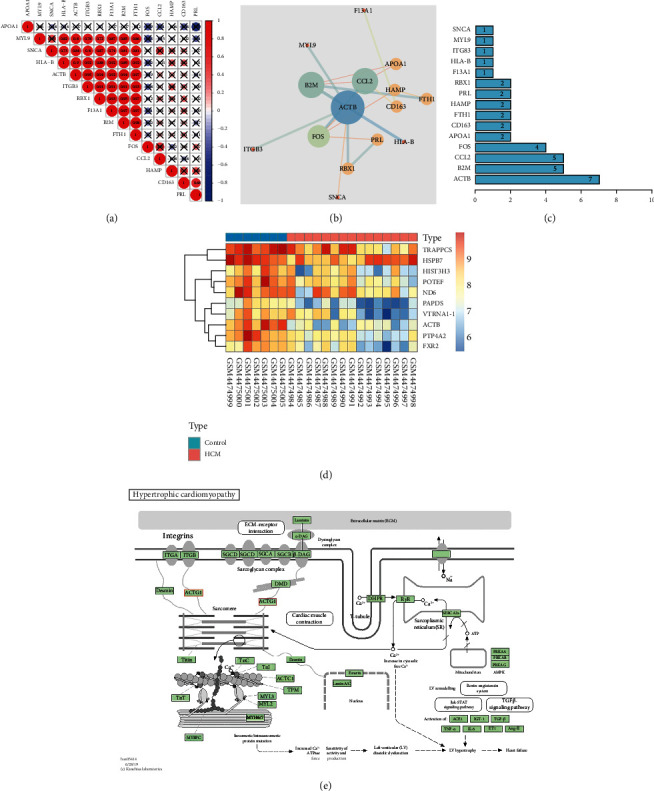
Key evidence for the involvement of ACTB in HCM. (a) The correlation between the expression of 15 candidate DEGs analyzed by GSE148602 dataset. (b) Interaction network diagram of 15 candidate DEGs (nodes represent proteins, and edges represent interrelationships between proteins. The larger the circle, the greater the degree value. The color of the circle changes from red to blue, indicating that the degree value changes from small to large, and the color of the line changes from red to blue, indicating that the combine score value changes from small to large). (c) 15 candidate DEGs sorted by degree. (d) ACTB expression in plasma of HCM patients and normal controls analyzed using GSE148602 dataset. (e) The specific regulatory relationship of HCM signaling pathway (hsa05414) analyzed by KEGG.

Correlation analyses of the expression levels of the 15 candidate DEGs using the expression data in the GSE148602 dataset revealed that MYL9, SNCA, HLA-B, ACTB, ITGB3, RBX1, F13A1, B2M, FTH1, and FOS were significant expressed (Figure 2(a)). Subsequently, the 15 candidate DEGs were imported into the STRING database with the species limited as human to obtain the protein interaction relationship and then imported into the Cytoscape software to construct a protein-protein interaction (PPI) network. The PPI network relationship graph included 15 nodes and 19 edges (PPI enrichment *P* value < 7.44*e*-05). The larger the shape, the greater the degree value, while the greater the degree value corresponding to the color changing from red to blue, the higher the combine score value (Figure 2(b)). Degree was indicative of the number of connections between a node and other nodes in the regulatory network, that is, the core degree. According to the degree value, it was found that ACTB ranked first (Figure 2(c)). Thereafter, the GSE148602 dataset was retrieved to analyze ACTB expression, which demonstrated that ACTB was poorly expressed in the plasma of HCM patients relative to normal healthy controls (Figure 2(d)), which was suggestive of the potential role ACTB as a circulation marker of HCM. Additionally, KEGG pathway analysis results indicated that ACTB played an important role in the hypertrophic cardiomyopathy signaling pathway (hsa05414) (Figure 2(e)). Altogether, that ACTB may serve as a key gene involved in occurrence of HCM.

### 3.3. Prediction of Upstream miRNA, lncRNA, and circRNA of ACTB

**Figure 3:**
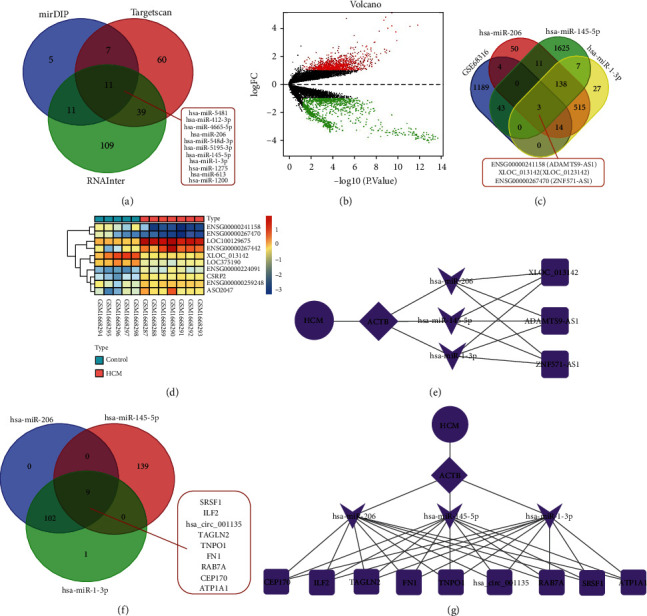
Screening of upstream miRNA, lncRNA, and circRNA of ACTB. (a) Venn diagram of screening results from mirDIP, TargetScan, and RNAInter databases. (b) The differentially expressed mRNA in the HCM samples and the normal samples in the GSE68316 dataset. Green dots indicate downregulation, red dots indicate upregulation, gray dots indicate no significant difference, and the horizontal axis represents the logarithmic value of the multiple (FC) of the difference between different groups at the base of 2, log_2_ (FC), and the vertical axis represents the negative logarithmic value of the *P* value for the significance test of the difference, which was -log10 (*P* value). (c) Venn diagram of the intersection of the prediction results of DIANA-LncBase and the downregulated lncRNAs in the GSE68316 dataset. (d) Heat map of the GSE68316 dataset. (e) The regulatory network of lncRNA-miRNA-ACTB plotted by the Cytoscape software. (f) Venn diagram of StarBase database prediction results. (g) The regulatory network of circRNA-miRNA-ACTB plotted by the Cytoscape software.

Accumulating evidence has reported the involvement of the lncRNA-miRNA-mRNA axis in cardiovascular diseases [8], with some even possessing great potential to serve as biomarkers. Accordingly, we adopted 3 common online databases (mirDIP, TargetScan, and RNAInter) to predict the upstream miRNA of ACTB with the species limited as human. The mirDIP database predicted 34 miRNAs (integrated score > 0.30), the TargetScan database predicted 129 miRNAs (context + +score < −0.2), and the RNAInter database predicted 170 miRNAs (score > 0.5). Subsequently, the obtained results from the above three databases were intersected with the 11 obtained miRNAs (Figure 3(a)). Among them, miR-206, miR-145-5p, and miR-1-3p exhibited a good binding relationship with ACTB (Table S2). A number of studies have elaborated the functional role of miR-206 in cardiac hypertrophy [10, 18–20], Meanwhile, miR-145-5p is also known to differentially express in primary dilated cardiomyopathy [21], while also exhibiting a certain relationship with myocardial ischemia-reperfusion injury [22]. Moreover, inhibition of miR-145-5p was previously shown to protect cardiac dysfunction after cardiac arrest and resuscitation [23]. Lastly, studies have also documented increased expressions of miR-1-3p in peripheral blood of patients with acute viral myocarditis and further associated with myocardial injury [24], left ventricular end diastolic diameter (LVEDD), and left ventricular ejection fraction (LVEF) reflecting cardiac function of HCM [25]. Furthermore, evidence also suggests miR-1-3p is also associated with hypertrophy and fibrosis in HCM [26].

Afterwards, we employed the DIANA-LncBase database to predict the upstream lncRNAs of miR-206, miR-145-5p, and miR-1-3p (screening condition was score > 0.7), which reared a total of 735, 1827, and 704 lncRNAs, respectively. Meanwhile, 193 differentially expressed lncRNAs were also identified from the HCM-related dataset GSE68316, wherein 67 were upregulated and 126 were downregulated (Figure 3(b)). Subsequent intersection of the DIANA-LncBase and the downregulated lncRNAs in GSE68316 dataset revealed 3 candidate lncRNAs, namely, ENSG00000241158 (ADAMTS9-AS1), XLOC_013142 (XLOC_013142), and ENSG00000267470 (ZNF571-AS1) (Figure 3(c)). The specific expression patterns and the combination relationship are shown in Figure 3(d) and Table S3. The Cytoscape software was then adopted to draw the network regulation diagram and construct the lncRNA-miRNA-ACTB coexpression regulation network (Figure 3(e)).

Furthermore, the StarBase database was employed to predict the upstream circRNAs of miR-206, miR-145-5p, and miR-1-3p (the screening conditions were genome = human, clipExpNum > 10), with 111, 148, and 103 circRNAs obtained, respectively. Following intersection, 9 candidate circRNAs (SRSF1, ILF2, CIRC_001135, TAGLN2, TNPO1, FN1, RAB7A, CEP170, and ATP1A1) were obtained (Figure 3(f)). Table S4 illustrates the specific combination relationship. Subsequently, the Cytoscape software was adopted to draw the network regulation diagram and construct the circRNA-miRNA-ACTB regulation network (Figure 3(g)).

### 3.4. ADAMTS9-AS1/miR-206/ACTB and circFN1/miR-206/ACTB Pathways Participate in the Occurrence of HCM

**Figure 4:**
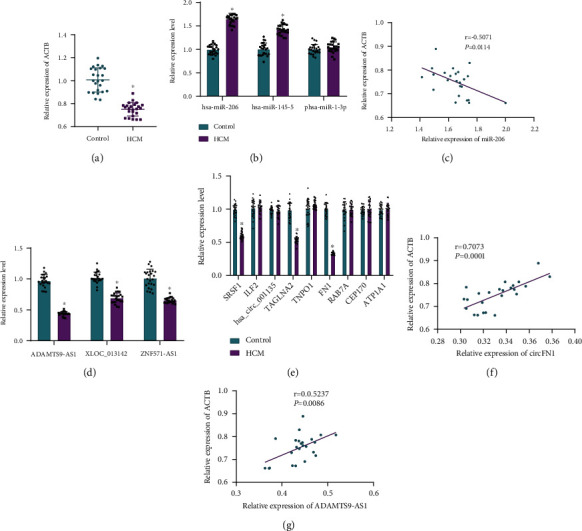
Expression of ACTB and its upstream miRNA, lncRNA, and circRNA in peripheral blood of patients with HCM. (a) mRNA expression of ACTB in peripheral blood samples of patients with HCM and normal healthy controls detected using RT-qPCR. (b) miRNA expression in peripheral blood samples of HCM patients and normal healthy controls measured using RT-qPCR. (c) The correlation between miR-206 and ACTB analyzed by Pearson. (d) lncRNA expression in peripheral blood samples of HCM patients and normal healthy controls determined by RT-qPCR. (e) circRNA expression in peripheral blood samples of HCM patients and normal healthy controls determined by RT-qPCR. (f) The correlation between circFN1 and ACTB analyzed by Pearson. (g) The correlation between ADAMTS9-AS1 and ACTB analyzed by Pearson. Measurement data were expressed by mean ± standard deviation. Independent sample *t* test was conducted for comparisons between two groups. ANOVA was conducted for multiple group comparison, followed by Tukey's post hoc test. Pearson correlation was used to analyze the correlation between circFN1 and ACTB, ADAMTS9-AS1 and ACTB, and miR-206 and ACTB. ^∗^*P* < 0.05 vs. normal healthy controls.

To further elucidate the mechanism of ACTB and its upstream miRNA, lncRNA, and circRNA in occurrence of HCM, as well as their value as markers of blood circulation, the peripheral blood samples of HCM patients and normal healthy controls were collected. Subsequent RT-qPCR analyses showed that ACTB expression levels were decreased in the peripheral blood of HCM patients compared with those in normal healthy controls (Figure 4(a)). We observed no significant differences in miR-1-3p expression levels, but miR-206 and miR-145-5p expression levels were increased, with miR-206 being the most profoundly expressed (Figure 4(b)). In addition, miR-206 was negatively correlated with ACTB expression (Figure 4(c)). Meanwhile, compared with normal healthy controls, ADAMTS9-AS1, XLOC_013142, ZNF571-AS1, SRSF1, TAGLN2, and FN1 expression levels were all downregulated in the peripheral blood of HCM patients. Among them, ADAMTS9-AS1 and circFN1 were significantly downregulated, while no significant difference was documented in expression of other circRNAs (Figures 4(d) and 4(e)). Simultaneously, circFN1 and ADAMTS9-AS1 were both positively correlated with ACTB (Figures 4(f) and 4(g)). Altogether, these findings indicated that the coexpression regulatory network of ADAMTS9-AS1/miR-206/ACTB and circFN1/miR-206/ACTB may serve as the key pathway involved in occurrence of HCM.

### 3.5. Both ADAMTS9-AS1 and circFN1 Can Competitively Bind miR-206, while miR-206 Can Target and Inhibit ACTB

**Figure 5:**
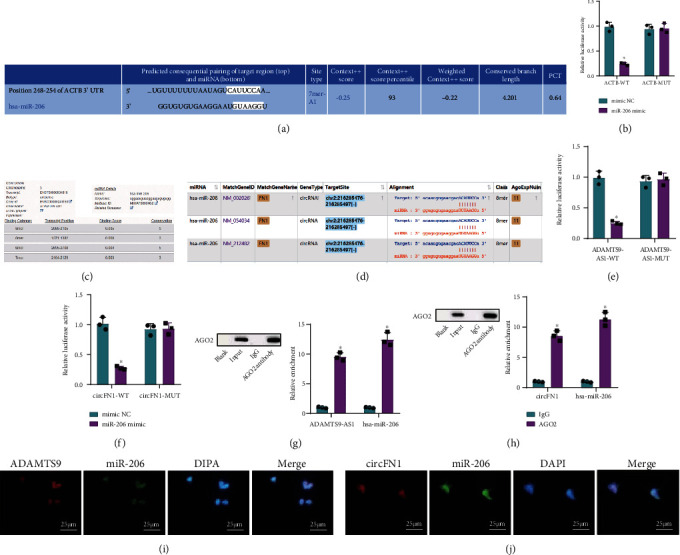
The targeted regulatory relationships between ADAMTS9-AS1/miR-206/ACTB and circFN1/miR-206/ACTB pathways. (a) Schematic diagram of the binding sites of miR-206 and ACTB predicted on the TargetScan website. (b) The results for miR-206 and ACTB verified by dual-luciferase reporter gene assay. (c) The binding sites of ADAMTS9-AS1 and miR-206 predicted using the DIANA-LncBase database. (d) The binding sites of circFN1 and miR-206 predicted using the Starbase database. (e) The results of ADAMTS9-AS1 and miR-206 verified by dual-luciferase reporter gene assay. (f) The results of circFN1 and miR-206 verified by dual-luciferase reporter gene assay. (g) Results of ADAMTS9-AS1 and miR-206 verified by RIP assay. (h) Results of circFN1 and miR-206 verified by RIP assay. (i) Results of ADAMTS9-AS1 and miR-206 observed by FISH. (j) Results of circFN1 and miR-206 observed by FISH. Measurement data were expressed by mean ± standard deviation. Independent sample *t* test was conducted for comparisons between two groups. ^∗^*P* < 0.05 vs. the mimic NC group or the IgG group.

The TargetScan database was adopted to predict the binding site of miR-206 and ACTB (Figure 5(a)). Subsequent dual-luciferase reporter gene assay confirmed that in HEK293 cells, the luciferase signal of cells after cotransfection of ACTCB-WT and miR-206-mimic was decreased compared with those transfected with ACTCB-WT and NC mimic. However, the luciferase signal of cells after cotransfection of ACTB-MUT showed no significant differences (Figure 5(b)). Overall, these findings indicated that miR-206 could target and inhibit ACTB expression.

To further explore the binding relationship between ADAMTS9-AS1, circFN1, and miR-206, we predicted the binding sites of ADAMTS9-AS1 and miR-206 and circFN1 and miR-206 with the help of the DIANA-LncBase and Starbase databases (Figures 5(c) and 5(d)). Dual-luciferase reporter gene assay further illustrated that in HEK293 cells, the luciferase signal of cells with ADAMTS9-AS1-WT/circFN1-WT and miR-206-mimic cotransfection was decreased compared to those cotransfected with ADAMTS9-AS1-WT/circFN1-WT and NC mimic (*P* < 0.05), while ADAMTS9-AS1-MUT and circFN1-MUT exhibited no significant differences in luciferase signal (*P* > 0.05) (Figures 5(e) and 5(f)). These findings suggested that both ADAMTS9-AS1 and circFN1 could competitively bind to miR-206.

Existing evidence further suggests that miRNAs possess the ability to bind to MREs through RNA-induced silencing complex, of which AGO2 protein is the key component [27]. We further adopted RIP to detect the binding of ADAMTS9-AS1/circFN1 and miR-206 to AGO2 protein, which revealed that ADAMTS9-AS1 and circFN1 could both bind to the AGO of miR-206 (Figures 5(g) and 5(h)). Moreover, RNA FISH illustrated that ADAMTS9-AS1/circFN1 and miR-206 appeared in the same position in the cell (Figures 5(i) and 5(j)). Altogether, the above results illustrated that ADAMTS9-AS1 and circFN1 could competitively bind to miR-206.

## 4. Discussion

Despite the tremendous advances made to uncover the diverse gene mutation sites in HCM, the molecular pathway from genotype to phenotype remains unclear, while the regulatory mechanism in the occurrence and development of cardiac hypertrophy also require much elaboration [28]. Nevertheless, the hard-done work of our peers has shed a light on the critical functions of lncRNA/circRNA-miRNA-mRNA networks in cardiovascular diseases [29, 30]. Accordingly, the current study sets out to investigate the possible molecular mechanism of the lncRNA/circRNA-miRNA-mRNA coexpression regulatory network implicated in the occurrence of HCM. Ultimately, our findings revealed that ADAMTS9-AS1/circFN1 competitively bound to miR-206 to elevate ACTB expression, which confers an inducive effect on HCM progression.

Initial findings in our study suggested that ACTB might serve as a key gene in the emergence of HCM. Subsequent analyses further revealed that ACTB was poorly expressed in plasma from patients with HCM. Existing evidence suggests that HCM is featured by interstitial fibrosis, asymmetrical left ventricular hypertrophy, and diastolic dysfunction, which often augment the risk of sudden cardiac death at an early age [31]. Moreover, prior studies have documented hundreds of mutations in the genes encoding the protein components of the sarcomere, all of which increase the sensitivity of myocardial filaments to calcium ions in HCM [32]. In addition, the sensitivity of myosin to Ca2^+^ increases, and the use of ATP by actomyosin also increases at submaximum Ca2^+^ concentrations, which can precipitate an imbalance between cardiac energy supply and demand under severe stress [28]. Meanwhile, the actin encoded by the ACTB gene is a highly conserved protein that aggregates in the cell cytoplasm to produce fibers and form a cross-linked network [33]. Interestingly, a large amount of literature has evidenced the differential expressions of ACTB in a variety of cardiovascular diseases [6, 34]. Additionally, we observed that ADAMTS9-AS1 and circFN1 expressions were significantly decreased, while those of miR-206 were markedly increased in HCM. Furthermore, out finding validated that miR-206 was the upstream of ACTB, while ADAMTS9-AS1 and circFN1 served as the upstream of miR-206. It is also noteworthy that numerous studies have implicated the lncRNA-miRNA-mRNA network in various cardiovascular diseases, while miRNA, lncRNA, and circRNA are also known to exhibit certain value as blood circulation markers [35, 36]. For instance, a prior study illustrated that the content of circRNA MICRA was low in the peripheral blood of patients with myocardial infarction, whereas poor MICRA levels are associated with a high risk of left ventricular dysfunction [37, 38]. On the other hand, plasma levels of miR-208a were previously indicated as a biomarker of human acute myocardial infarction [39]. Similarly, circulating miRNAs in HCM, such as miR-29a, have also indicated to function as biomarkers of HCM hypertrophy and fibrosis [40]. In addition, another study suggested that miR-206 exhibits a profound effect on a series of myopathies, including hypertrophy, malnutrition, and conduction defects [41]. Besides, hsa-miR-206 is differentially expressed in patients with arrhythmic right ventricular cardiomyopathy [42]. Strikingly, a previous study highlighted that inhibition of miR-206 could improve mouse ischemia-reperfusion in arrhythmia models by targeting connexin 43 [43]. Lastly, there is also evidence to suggest that multiple circRNAs are differently expressed in different tissues and peripheral blood [44]. In addition, circRNAs or lncRNAs exhibit a subclass of noncoding RNAs and further possess the ability to regulate gene expression at a molecular level in different ways, e.g., functioning as miRNA sponges or regulating transcription and exerting important roles in multibiological processes, thus resulting in several kinds of diseases, including HCM [45–49]. FN1 has been identified to play an important role in myocardial fibrosis, contributing to identifying the etiology of dilated cardiomyopathy [16]. Meanwhile, despite the lack of research focusing on the role of ADAMTS9-AS1 in HCM, ADAMTS9-AS1 is known to be downregulated in other cancers such as prostate cancer and colorectal cancer [50, 51]. Besides, ADAMTS9-AS1 was shown to be poorly expressed in breast cancer tissues and cells, whereas upregulation of ADAMTS9-AS1 could induce inhibited cell growth and invasiveness both *in vivo and in vitro* by attenuating miR-513a-5p expression [14]. All of the aforementioned findings and evidences shed a new light on the potential function of the regulatory network in the occurrence of HCM.

## 5. Conclusions

**Figure 6:**
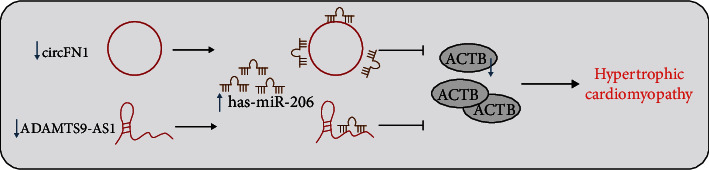
Schematic diagram of the molecular mechanism of ADAMTS9-AS1/miR-206/ACTB and circFN1/miR-206/ACTB regulatory network involved in the occurrence of HCM.

Taken together, our findings indicate that that ADAMTS9-AS1 and circFN1 may act as ceRNA to competitively bind miR-206 to upregulate ACTB in HCM. In addition, it would be prudent to suggest that ADAMTS9-AS1, circFN1, miR-206, and ACTB have the potential to serve as potential blood circulation markers for HCM (Figure 6). However, the project lacks clinical tissue sample verifications. At the same time, clinical blood samples and patient clinical data should be further expanded, so as to reverify the clinical predictive value of ceRNA regulatory network, which will be the focus of our future endeavors. Overall, we hope our discoveries provide a theoretical basis for an in-depth understanding of the HCM mechanism and augment the search for potential biomarkers.

## Data Availability

The datasets generated/analyzed during the current study are available.

## Supplementary Materials

Supplementary Materials Figure S1: flow chart of bioinformatics screening of key genes for HCM. Table S1: primer sequences for RT-qPCR. Note: ACTB: beta-actin; ADAMTS9-AS1: long noncoding RNA ADAMTS9-antisense 1; ZNF571-AS1: long noncoding RNA ZNF571-antisense 1; GAPDH: glyceraldehyde-3-phosphate dehydrogenase. Table S2: comparison of the targeting relationship between 11 miRNAs and ACTB. Table S3: binding relationship between lncRNA and miRNA. Table S4: binding relationship between circRNA and miRNA.

## References

[1] Tuohy C. V., Kaul S., Song H. K., Nazer B., Heitner S. B. (2020). Hypertrophic cardiomyopathy: the future of treatment. *European Journal of Heart Failure*.

[2] Huang Y., Lu H., Ren X. (2020). Fropofol prevents disease progression in mice with hypertrophic cardiomyopathy. *Cardiovascular Research*.

[3] Maron B. J., Rowin E. J., Maron M. S. (2019). Paradigm of sudden death prevention in hypertrophic cardiomyopathy. *Circulation Research*.

[4] Wijnker P. J. M., Sequeira V., Kuster D. W. D., Velden J. V. (2019). Hypertrophic cardiomyopathy: a vicious cycle triggered by sarcomere mutations and secondary disease hits. *Antioxidants & Redox Signaling*.

[5] Yang S., Zhao Y., Chen X. (2020). The ACTB variants and alcohol drinking confer joint effect to ischemic stroke in Chinese Han population. *Journal of Atherosclerosis and Thrombosis*.

[6] Xue J., Zhou D., Poulsen O. (2018). Exploring miRNA-mRNA regulatory network in cardiac pathology in Na^+^/H^+^ exchanger isoform 1 transgenic mice. *Physiological Genomics*.

[7] Zhu X., Zhang L., Hu Y., Zhang J. (2018). Identification of suitable reference genes for real-time qPCR in homocysteine-treated human umbilical vein endothelial cells. *PLoS One*.

[8] Huang Y. (2018). The novel regulatory role of lncRNA-miRNA-mRNA axis in cardiovascular diseases. *Journal of Cellular and Molecular Medicine*.

[9] Li M., Duan L., Li Y., Liu B. (2019). Long noncoding RNA/circular noncoding RNA-miRNA-mRNA axes in cardiovascular diseases. *Life Sciences*.

[10] Yang Y., del Re D. P., Nakano N. (2015). miR-206 mediates YAP-induced cardiac hypertrophy and survival. *Circulation Research*.

[11] Obradovic D., Rommel K. P., Blazek S. (2021). The potential role of plasma miR-155 and miR-206 as circulatory biomarkers in inflammatory cardiomyopathy. *ESC Heart Failure*.

[12] Huarte M. (2015). The emerging role of lncRNAs in cancer. *Nature Medicine*.

[13] Wan J., Jiang S., Jiang Y. (2019). Data mining and expression analysis of differential lncRNA ADAMTS9-AS1 in prostate cancer. *Frontiers in Genetics*.

[14] Fang S., Zhao Y., Hu X. (2020). LncRNA ADAMTS9-AS1 restrains the aggressive traits of breast carcinoma cells via sponging miR-513a-5p. *Cancer Management and Research*.

[15] Zhang Z., Li H., Hu Y., Wang F. (2020). Long non-coding RNA AdAMTS9-AS1 exacerbates cell proliferation, migration, and invasion via triggering of the PI3K/AKT/mTOR pathway in hepatocellular carcinoma cells. *American Journal of Translational Research*.

[16] Huang K., Wen S., Huang J. (2020). Integrated analysis of hub genes and mirnas in dilated cardiomyopathy. *BioMed Research International*.

[17] Wang X., Chen X. X., Yu H. T. (2020). Engineered cardiac tissues: a novel in vitro model to investigate the pathophysiology of mouse diabetic cardiomyopathy. *Acta Pharmacologica Sinica*.

[18] Kong F., Jin J., Lv X. (2019). RETRACTED: Long noncoding RNA RMRP upregulation aggravates myocardial ischemia-reperfusion injury by sponging miR-206 to target ATG3 expression. *Biomedicine & Pharmacotherapy*.

[19] Li T., Yu S. S., Zhou C. Y., Wang K., Wan Y. C. (2020). MicroRNA-206 inhibition and activation of the AMPK/Nampt signalling pathway enhance sevoflurane post-conditioning-induced amelioration of myocardial ischaemia/reperfusion injury. *Journal of Drug Targeting*.

[20] Zhai C., Qian Q., Tang G. (2017). MicroRNA-206 protects against myocardial ischaemia-reperfusion injury in rats by targeting Gadd45*β*. *Molecules and Cells*.

[21] Toro R., Blasco-Turrión S., Morales-Ponce F. J. (2018). Plasma microRNAs as biomarkers for Lamin A/C-related dilated cardiomyopathy. *Journal of Molecular Medicine (Berlin, Germany)*.

[22] Chen G., Xu C., Gillette T. G. (2020). Cardiomyocyte-derived small extracellular vesicles can signal enos activation in cardiac microvascular endothelial cells to protect against ischemia/reperfusion injury. *Theranostics*.

[23] He Y., Wang G., Li C., Wang Y., Zhang Q. (2021). The protective effects of phosphodiesterase-5 inhibitor, sildenafil on post-resuscitation cardiac dysfunction of cardiac arrest: by regulating the miR-155-5p and miR-145-5p. *Scandinavian Journal of Trauma, Resuscitation and Emergency Medicine*.

[24] Marketou M., Kontaraki J., Patrianakos A. (2021). Peripheral blood micrornas as potential biomarkers of myocardial damage in acute viral myocarditis. *Genes*.

[25] Li M., Chen X., Chen L., Chen K., Zhou J., Song J. (2018). MiR-1-3p that correlates with left ventricular function of HCM can serve as a potential target and differentiate HCM from DCM. *Journal of Translational Medicine*.

[26] Scolari F. L., Faganello L. S., Garbin H. I., Piva E. M. B., Biolo A. (2021). A systematic review of microRNAs in patients with hypertrophic cardiomyopathy. *International Journal of Cardiology*.

[27] Ikeda K., Satoh M., Pauley K. M., Fritzler M. J., Reeves W. H., Chan E. K. L. (2006). Detection of the argonaute protein Ago2 and microRNAs in the RNA induced silencing complex (RISC) using a monoclonal antibody. *Journal of Immunological Methods*.

[28] Maron B. J., Maron M. S. (2013). Hypertrophic cardiomyopathy. *Lancet*.

[29] Su Q., Lv X. (2020). Revealing new landscape of cardiovascular disease through circular RNA-miRNA-mRNA axis. *Genomics*.

[30] Song C., Zhang J., Qi H. (2017). The global view of mRNA-related ceRNA cross-talks across cardiovascular diseases. *Scientific Reports*.

[31] Wijnker P. J. M., van der Velden J. (2020). Mutation-specific pathology and treatment of hypertrophic cardiomyopathy in patients, mouse models and human engineered heart tissue. *Biochimica et Biophysica Acta - Molecular Basis of Disease*.

[32] Pablo Kaski J., Syrris P., Shaw A. (2012). Prevalence of sequence variants in the RAS-mitogen activated protein kinase signaling pathway in pre-adolescent children with hypertrophic cardiomyopathy. *Circulation Cardiovascular Genetics*.

[33] Drazic A., Aksnes H., Marie M. (2018). NAA80 is actin's N-terminal acetyltransferase and regulates cytoskeleton assembly and cell motility. *Proceedings of the National Academy of Sciences of the United States of America*.

[34] Wang W., Liu Q., Wang Y. (2019). Integration of gene expression profile data of human epicardial adipose tissue from coronary artery disease to verification of hub genes and pathways. *BioMed Research International*.

[35] Wang G., Zheng X., Zheng Y. (2019). Construction and analysis of the lncRNA‑miRNA‑mRNA network based on competitive endogenous RNA reveals functional genes in heart failure. *Molecular Medicine Reports*.

[36] Tao L., Shi J., Huang X., Hua F., Yang L. (2020). Identification of a lncRNA-miRNA-mRNA network based on competitive endogenous RNA theory reveals functional lncRNAs in hypertrophic cardiomyopathy. *Experimental and Therapeutic Medicine*.

[37] Vausort M., Salgado-Somoza A., Zhang L. (2016). Myocardial infarction-associated circular RNA predicting left ventricular dysfunction. *Journal of the American College of Cardiology*.

[38] Salgado-Somoza A., Zhang L., Vausort M., Devaux Y. (2017). The circular RNA MICRA for risk stratification after myocardial infarction. *IJC Heart & Vasculature*.

[39] Wang G. K., Zhu J. Q., Zhang J. T. (2010). Circulating microRNA: a novel potential biomarker for early diagnosis of acute myocardial infarction in humans. *European Heart Journal*.

[40] Roncarati R., Viviani Anselmi C., Losi M. A. (2014). Circulating miR-29a, among other up-regulated microRNAs, is the only biomarker for both hypertrophy and fibrosis in patients with hypertrophic cardiomyopathy. *Journal of the American College of Cardiology*.

[41] Townley-Tilson W. H., Callis T. E., Wang D. (2010). MicroRNAs 1, 133, and 206: critical factors of skeletal and cardiac muscle development, function, and disease. *The International Journal of Biochemistry & Cell Biology*.

[42] Khudiakov A. A., Panshin D. D., Fomicheva Y. V. (2021). Different expressions of pericardial fluid microRNAs in patients with arrhythmogenic right ventricular cardiomyopathy and ischemic heart disease undergoing ventricular tachycardia ablation. *Frontiers in cardiovascular medicine*.

[43] Jin Y., Zhou T., Feng Q. (2020). Inhibition of microRNA-206 ameliorates ischemia-reperfusion arrhythmia in a mouse model by targeting connexin43. *Journal of Cardiovascular Translational Research*.

[44] Werfel S., Nothjunge S., Schwarzmayr T., Strom T. M., Meitinger T., Engelhardt S. (2016). Characterization of circular RNAs in human, mouse and rat hearts. *Journal of Molecular and Cellular Cardiology*.

[45] Altesha M. A., Ni T., Khan A., Liu K., Zheng X. (2019). Circular RNA in cardiovascular disease. *Journal of Cellular Physiology*.

[46] Hansen T. B., Jensen T. I., Clausen B. H. (2013). Natural RNA circles function as efficient microRNA sponges. *Nature*.

[47] Sonnenschein K., Wilczek A. L., de Gonzalo-Calvo D. (2019). Serum circular RNAs act as blood-based biomarkers for hypertrophic obstructive cardiomyopathy. *Scientific Reports*.

[48] Liu X., Ma Y., Yin K. (2019). Long non-coding and coding RNA profiling using strand-specific RNA-seq in human hypertrophic cardiomyopathy. *Scientific data*.

[49] Hu X., Shen G., Lu X., Ding G., Shen L. (2019). Identification of key proteins and lncRNAs in hypertrophic cardiomyopathy by integrated network analysis. *Archives of Medical Science*.

[50] Zhou Z., Wu X., Zhou Y., Yan W. (2021). Long non-coding RNA ADAMTS9-AS1 inhibits the progression of prostate cancer by modulating the miR-142-5p/CCND1 axis. *The Journal of Gene Medicine*.

[51] Li N., Li J., Mi Q. (2020). Long non-coding RNA ADAMTS9-AS1 suppresses colorectal cancer by inhibiting the Wnt/*β*‐catenin signalling pathway and is a potential diagnostic biomarker. *Journal of Cellular and Molecular Medicine*.

