# Supplementary material for: lncRNA ADAMTS9-AS1/circFN1 Competitively Binds to miR-206 to Elevate the Expression of ACTB, Thus Inducing Hypertrophic Cardiomyopathy
Source: Oxid Med Cell Longev. 2022 Mar 31;2022:1450610. doi: 10.1155/2022/1450610 (PMC8989615; doi:10.1155/2022/1450610)
Supplement: Supplementary Materials — Figure S1: flow chart of bioinformatics screening of key genes for HCM. Table S1: primer sequences for RT-qPCR. Note: ACTB: beta-actin; ADAMTS9-AS1: long noncoding RNA ADAMTS9-antisense 1; ZNF571-AS1: long noncoding RNA ZNF571-antisense 1; GAPDH: glyceraldehyde-3-phosphate dehydrogenase. Table S2: comparison of the targeting relationship between 11 miRNAs and ACTB. Table S3: binding relationship between lncRNA and miRNA. Table S4: binding relationship between circRNA and miRNA. [file 1450610.f1.docx]

**
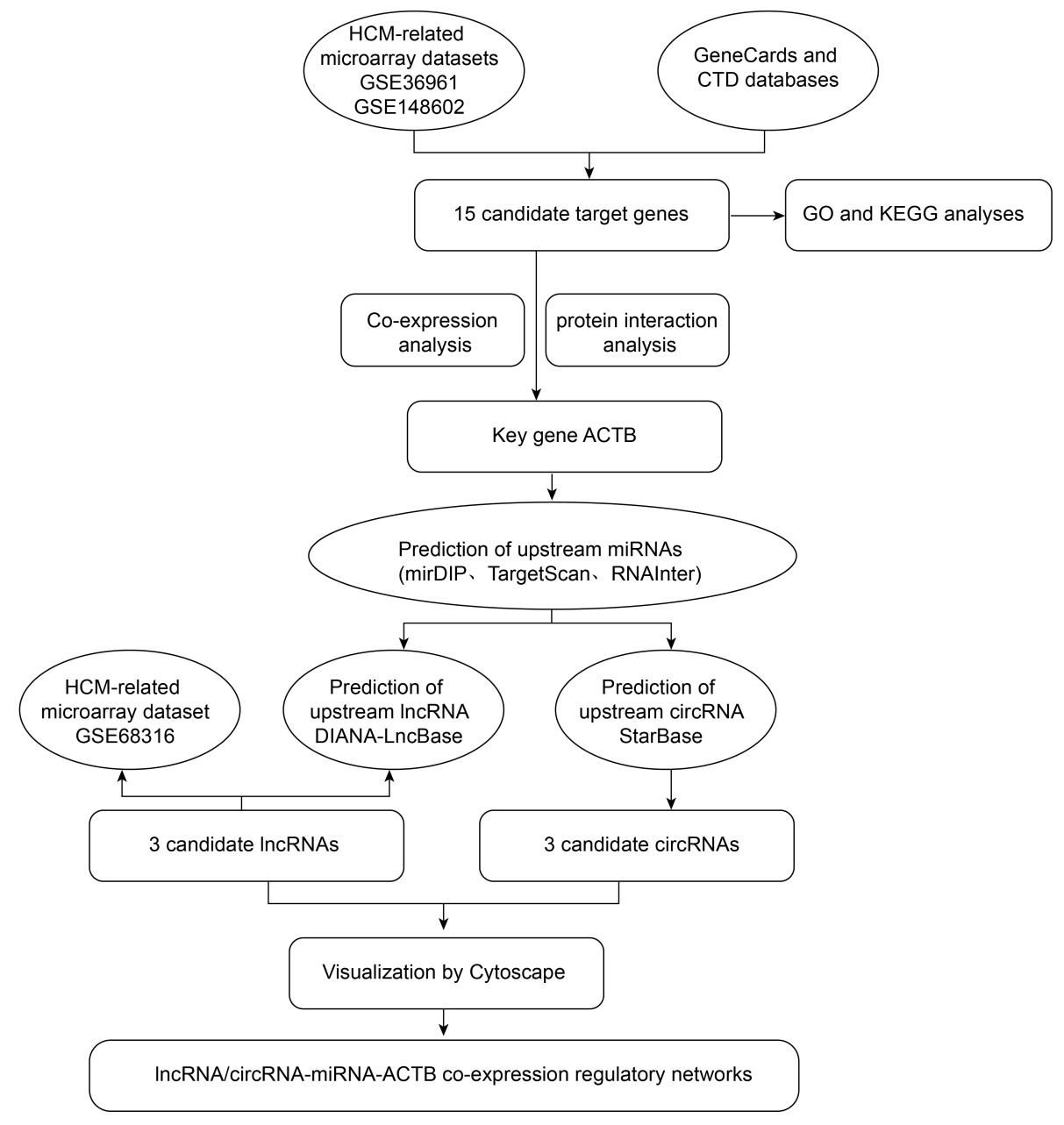
**

**FIGURE S1:** Flow chart of bioinformatics screening of key genes for HCM.

**TABLE S1:** Primer sequences for RT-qPCR

| Name | Primer sequence (5’-3’) |
| --- | --- |
| ACTB | Forward: CTCCATCCTGGCCTCGCTGT |
|  | Reserve: GCTGTCACCTTCACCGTTCC |
| hsa-miR-206 | Forward: ATGCACAAAAACAGCAGCAG |
|  | Reserve: GAAAAACCTTTGGGGGAAAG |
| miR-145-5p | Forward: CGCCAGAGGGTTTCCGGTACTTT |
|  | Reserve: CATCCAGCCTCACAGGGATGTTA |
| hsa-miR-1-3p | Forward: UGGAAUGUAAAGAAGUAUGUAU |
|  | Reserve: ACAUAC UUC UUUACAUUCCAUU |
| ADAMTS9-AS1 | Forward: TACTGGTTTGGACATGAGG |
|  | Reserve: AAAGGGGTGTTGGCACTC |
| XLOC_013142 | Forward: TCCCAGCTCTGACCCTCTTT |
|  | Reserve: CAGCACACTGGGGACAGTAG |
| ZNF571-AS1 | Forward: TGGCTCACAGCCCACAAAAT |
|  | Reserve: TGGGCATGCCTCTGTTCTAC |
| SRSF1 (hsa_circ_0044757) | Forward: AGAAGCGGAAGGAAACAGCG |
|  | Reserve: TGAACCCTGACCAGAAGTTGC |
| ILF2 (hsa_circ_0014268) | Forward: AGAGGCGTCTTGCCCTGTAT |
|  | Reserve: TGCCATTTTGATGGCACACCT |
| circ_001135 (hsa_circ_0001337) | Forward: GACAAGAATGACCGGGCCAA |
|  | Reserve: GCTCCACAGCAGAGAGGCTTA |
| TAGLN2 (hsa_circ_0014859) | Forward: GCACTGACTCAAGGCAAGGG |
|  | Reserve: CAGTGCCGAAAGGATGTGGG |
| TNPO1 (hsa_circ_0129555) | Forward: GGTCTCACGCTGTTGCATGT |
|  | Reserve: CCTGATTGGCTTCCAGATTGCT |
| FN1 (hsa_circ_0119099) | Forward: AGGAAGCCGAGGTTTTAACTG |
|  | Reserve: AGGAAGCCGAGGTTTTAACTG |
| RAB7A (hsa_circ_0067232) | Forward: GGGTGTGGGCTGAGTTCTTC |
|  | Reserve: GGTTCCAGGGAACTCTCCCG |
| CEP170 (hsa_circ_0017231) | Forward: CTGCCCAGTTCCAACGATCC |
|  | Reserve: CTCTCTCCACGGTAAGGGGG |
| ATP1A1 (hsa_circ_0013675) | Forward: CACTCCCCCTCCCACTACTC |
|  | Reserve: ATCACGTCCAACATTATCGTTTTGA |
| GAPDH | Forward: GAAGGTGAAGGTCGGAGTC |
|  | Reserve: GAAGATGGTGATGGGATTTC |
| U6 | Forward: CTCGCTTCG GCAGCACA |
|  | Reserve: AACGCTTCACGAATTTGCGT |

Note: ACTB, Beta-actin; ADAMTS9-AS1, Long non-coding RNA ADAMTS9-antisense 1; ZNF571-AS1, Long non-coding RNA ZNF571-antisense 1; GAPDH, Glyceraldehyde-3-phosphate dehydrogenase.

**TABLE S2:** Comparison of the targeting relationship between 11 miRNAs and ACTB

| lncRNAs | mirDIP (Integrated Score) | TargetScan (context++ score) | RNAInter (Score) |
| --- | --- | --- | --- |
| miR-548l | 0.377052273 | -0.21 | 0.5117 |
| miR-412-3p | 0.388745701 | -0.23 | 0.5831 |
| miR-4665-5p | 0.401576103 | -0.53 | 0.5831 |
| **miR-206** | **0.637233586** | **-0.25** | **0.5978** |
| miR-548d-3p | 0.396647147 | -0.23 | 0.5117 |
| miR-5195-3p | 0.456364234 | -0.56 | 0.5978 |
| **miR-145-5p** | **0.806613258** | **-0.47** | **0.9623** |
| **miR-1-3p** | **0.750627029** | **-0.25** | **0.749** |
| miR-1275 | 0.531153596 | -0.43 | 0.5831 |
| miR-613 | 0.479025121 | -0.26 | 0.6048 |
| miR-1200 | 0.530187569 | -0.26 | 0.5831 |

**TABLE S3:** Binding relationship between lncRNA and miRNA

| lncRNA | DIANA-LncBase (score) | | |
| --- | --- | --- | --- |
|  | miR-206 | miR-145-5p | miR-1-3p |
| ENSG00000241158 (ADAMTS9-AS1) | 0.914 | 0.914 | 0.894 |
| XLOC_013142 (XLOC_013142) | 0.975 | 0.914 | 0.954 |
| ENSG00000267470 (ZNF571-AS1) | 0.917 | 0.918 | 0.918 |

**TABLE S4:** Binding relationship between circRNA and miRNA

| circRNA | StarBase (clipExpNum) | | |
| --- | --- | --- | --- |
|  | miR-206 | miR-145-5p | miR-1-3p |
| SRSF1 | 18 | 12 | 18 |
| ILF2 | 10 | 10 | 10 |
| circ_001135 | 14 | 11 | 14 |
| TAGLN2 | 33 | 28 | 33 |
| TNPO1 | 17 | 16 | 17 |
| FN1 | 11 | 11 | 11 |
| RAB7A | 14 | 11 | 14 |
| CEP170 | 21 | 21 | 21 |
| ATP1A1 | 19 | 15 | 19 |
